# Novel dual: rod plate system for EOS improves vertebral wedging and permits spinal growth

**DOI:** 10.1186/s13018-023-04094-9

**Published:** 2023-09-29

**Authors:** Yang Zheng, Jian Zhou, Chunlei Niu, Qibin Ye, Jiazhu Tang, Xinyang Wang, Guanjun Wang

**Affiliations:** 1 Department of Orthopaedics, Fourth Medical Center of PLA General Hospital, Fucheng Road No. 51, Beijing, People’s Republic of China; 2 National Clinical Research Center for Orthopedics Sports Medicine and Rehabilitation, Beijing, People’s Republic of China; 3 School of Life Sciences, Hainan University, Hainan, People’s Republic of China; 4 Department of Orthopaedics, Third Medical Center of PLA General Hospital, Beijing, People’s Republic of China; 5 Department of Joint Surgery, Affiliated Hospital of Jiangsu University, Zhenjiang, Jiangsu People’s Republic of China; 6 Department of Orthopaedics, Huangshan City People’s Hospital, Huangshan, Anhui People’s Republic of China

**Keywords:** Plate-rod system for scoliosis, Early-onset scoliosis, Apical vertebral wedge angles, Tail rod, Rectify scoliosis deformity

## Abstract

**Background:**

To determine whether single-stage, growth-friendly instrumentation with a plate-rod spinal system (PRSS) can substantially correct the deformity of EOS at surgery and continue to rectify the deformity throughout the growth period.

**Methods:**

An observational study of 35 children with EOS treated by PRSS between February 2000 and October 2010 during a mean follow-up of 72 months. The mean age at surgery was 7 years. X-rays were taken preoperatively and postoperatively and at each follow-up. The Cobb angle, the apical vertebral wedge angle (AVWA), remaining rod lengths, maximal thoracic kyphosis and total T1-S1 heights were measured and compared.

**Results:**

Thirty-one patients, 9 boys and 22 girls, with a mean age of 7 years were completed follow-up. The Cobb angle changed from 64° to 36° after initial surgery and 26° at the last follow-up. The mean AVWA was 15° postoperatively and 5° at the last follow-up. The mean rod tail reserve length decreased from 53 mm immediately after surgery to 12 mm at the last follow-up. The mean preoperative maximum thoracic kyphosis was 41° and changed to 35° and 30° postoperatively and at latest follow-up, respectively. The mean preoperative T1–S1 height for all 32 patients was 52 mm acute lengthening and 122 mm of lengthening by the end of follow-up, respectively.

**Conclusion:**

The PRSS provided immediate correction of most of the deformity at surgery and continued to rectify remaining scoliosis during the growth period. AVWA may be a useful method for monitoring the function of the PRSS in EOS.

## Introduction

Early-onset scoliosis (EOS) is a pathological curvature of the spine, varying etiology, presenting before the age of 10 years [1]. If left untreated, EOS may lead to increased disability and potentially life-threatening conditions such as respiratory insufficiency and pulmonary hypertension and a reduced life expectancy. When EOS cannot be controlled by serial casts or braces, growing rods are a treatment option. Single or dual growing rods may be used to correct the deformity. Available friendly growing rod techniques have included the Shilla growth guidance techniques [2], the vertical expandable prosthetic titanium ribs (VEPTRs) implant, magnetically controlled growing rods (MCGRs) and traditional dual growing rods (TDGRs) [3–5]. Depending on the instrumentation, treatment with growing rods may require periodic surgery to lengthen the rods as the child grows [6], followed by definitive spinal fusion at the end of the growth period. Although many new techniques have been described in the management of early onset spinal deformities in recent years and these techniques have been applied in large series, an ideal method maintains effective correction and without fusion has not been developed yet. A plate-rod spinal system, the plate-rod system for scoliosis (PRSS), was developed at the Peking Union Medical College (PUMC) Hospital in 1998 and has been applied for the corrective treatment of EOS [7]. The one-stage PRSS procedure can correct the deformity without fusion and maintain the correction during growth. By reversing the unequal distribution of stress across the spine, the PRSS may lessen or terminate asymmetric growth, allowing for enduring correction of scoliosis in children with EOS [7].

The progression of the vertebral wedging component of EOS is attributed to the mechanical modulation of vertebral growth, as described in the Hueter–Volkmann Law [8]. Scoliosis with vertebral wedging caused by asymmetric mechanical loading can be corrected by reversing the loading [8]. A number of studies in animal models has confirmed the connection between asymmetric loading and asymmetric bone growth [9–12]. Compressive stress inhibits chondrocyte proliferation in growing and developing plate cartilage [13]. As a result, vertebral growth is inhibited on the compressive stress side and is promoted on the tensile stress side. Axial mechanical loading affects spine growth and plays an important role in scoliosis progression [13, 14]. It is the reason why the degree of scoliosis can be progressive without intervention. Chronic asymmetry of static loading associated with expansion of the apical vertebral wedge angle (AVWA) can be corrected by reversing the loading. The vertebral wedging attenuated may involve in spinal deformity correction.

We have previously demonstrated that PRSS is an effective instrumentation for correcting scoliosis in growing children in the short term (average 2.8 years follow-up) [7]. Now, after mid-term follow-up in children with EOS corrected by PRSS, we have investigated the transformation of the AVWA and evaluated the efficacy and therapeutic mechanism of the PRSS in the treatment of EOS. We want to clarify the relationship between spinal deformity correction and AVWA.

## Materials and methods

Children with progressive thoracic or thoracolumbar EOS operated by the senior authors at the General Hospital of Chinese Armed Police Forces, Beijing, China, from February 2000 to October 2010 were enrolled. Intraoperative spinal cord monitoring with the recording of both somatosensory evoked potentials (SSEP) and motor evoked potentials (MEP) was used.

Thirty-five patients met the following inclusion criteria for this retrospective analysis of prospectively collected observational data. (1) EOS of idiopathic scoliosis with no congenital scoliosis, intrinsic bone disease or underlying condition such as skeletal dysplasia, neurofibromatosis and endocrine diseases; (2) If non-operative treatment, including bracing, casting and halo gravity traction (HGT), had certain limitations and curve progressed. The thoracolumbar angle was more than 40 degrees and thoracolumbar/lumbar angle was more than 35 degrees. Surgical interventions were usually indicated; (3) Underwent the surgical PRSS procedure. All patients were followed for a minimum of 24 months after surgery, and the average follow-up was 72 months (range, 24–108 months).

Preoperative patient planning involves a meticulous evaluation of upright coronal and sagittal X-rays, as well as an analysis of the curve's flexibility through bend X-rays. It is crucial to accurately identify the location of the end vertebrae, stable vertebra and apical vertebral segments. The main principle of surgical fixation is to stabilize the upper portion to the upper end vertebra and the lower portion to the stable vertebra.

**Fig. 1:**
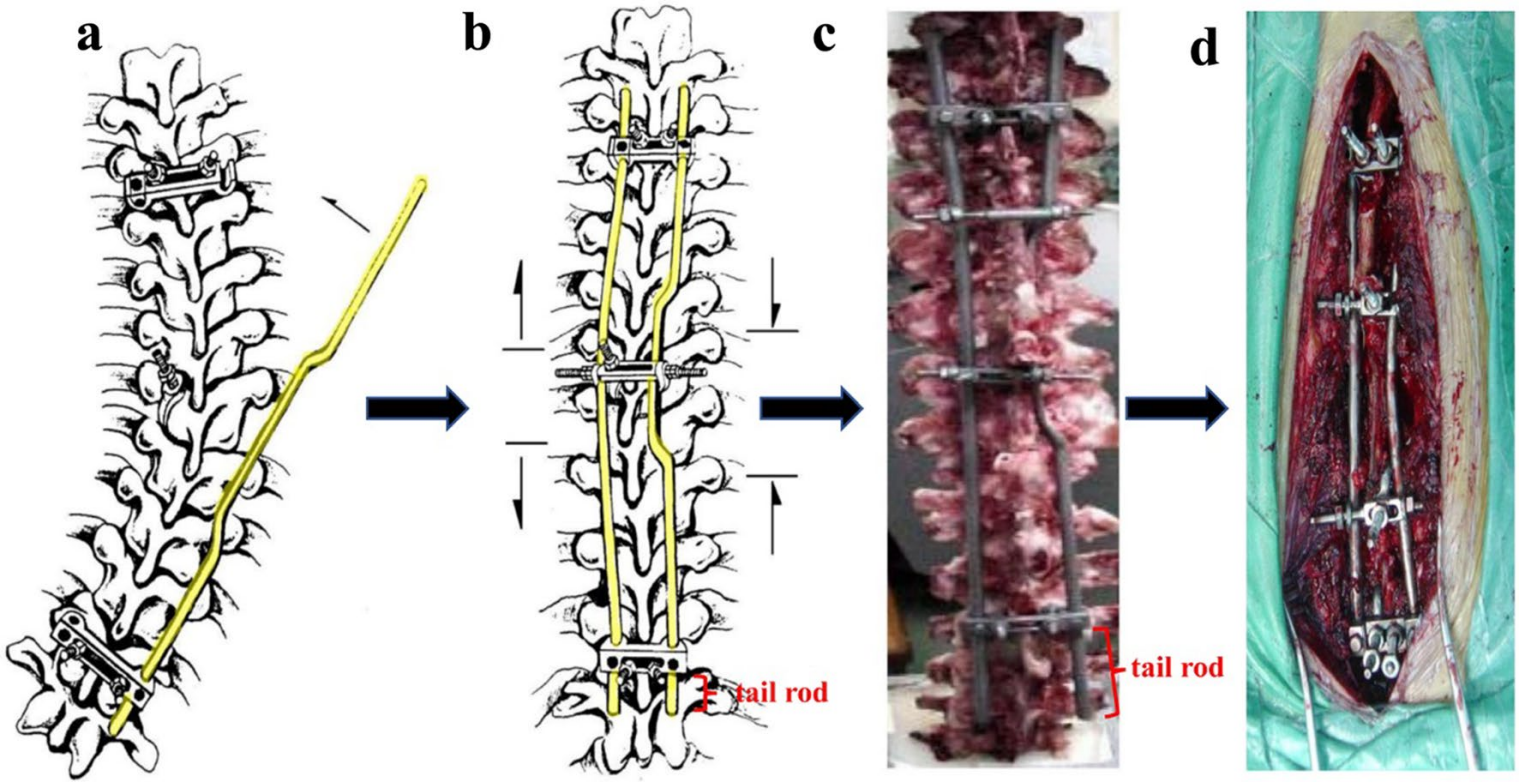
Asymmetrical stress is created by the lateral sidewise push of the plate-rod assembly. The PRSS can be seen as an inner brace. The lower part of each rod (tail rods) is able to move freely within the insertion point in the lower connector, allowing the rods to keep pace with the lengthening of the vertebral column during normal growth

During PRSS surgery screw hooks are placed under the lamina and fixed to the lamina with a screw to minimize the risk of dislodgment. The screws at the end vertebral segment are linked with the end connectors. Plates and cylindrical rods are connected by upper and lower connectors, thus forming a strong frame-like structure. Corrective force is provided by the lateral sidewise push of the plate rod (Fig. 1). There is no additional distractive force during surgical correction, which minimizes the risk of cord damage caused by overcorrection. The PRSS is a type of sliding instrumentation. Only the upper end of the device is tightly fixed in place with the set screw in the upper connecter, while the lower ends of the rods (tail rods) are intended to slide freely within the insertion points in the lower connector, allowing for gradual upward migration of the rods that keeps up with the longitudinal growth of the instrumented spinal segments and obviates the need for repeated operations. No bony fusion was performed [7].

**Fig. 2:**
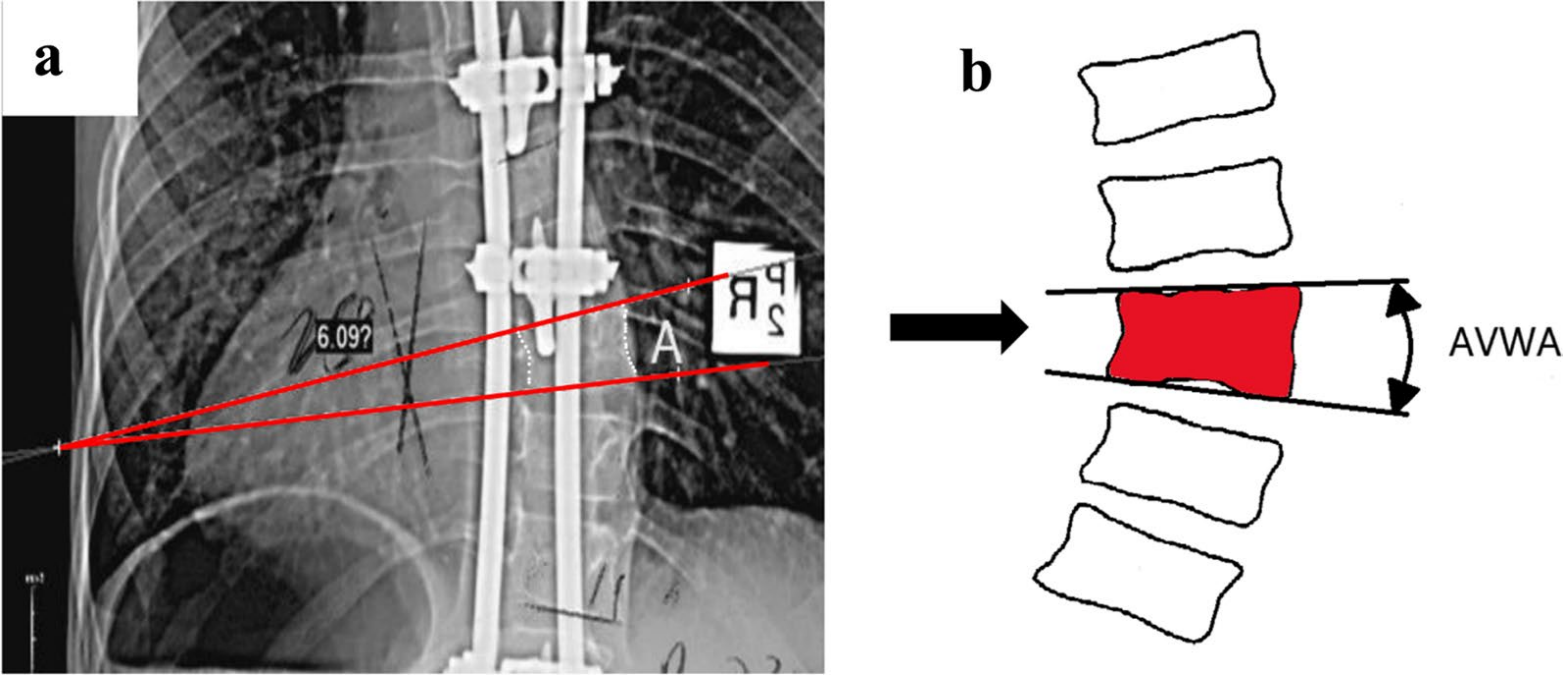
Measurement of the apical vertebral wedge angle. **a** The apical vertebra (A) on plain radiograph. **b** Schematic representation. AVWA, apical vertebral wedge angle

Total spine X-ray was performed in all patients preoperatively and postoperatively and at each follow-up visit, and the Cobb angle, AVWA, remaining rod lengths, maximal thoracic kyphosis and total T1-S1 heights were measured and recorded electronically by using Mimics 10.0 software. The apical vertebra was defined as the vertebra with the maximum lateral deviation from the patient’s spinal axis, as selected by the software. The AVWA was measured on the P-A radiographs by drawing lines across the superior and inferior endplates of the apical vertebra and measuring the angle between the two lines (Fig. 2) [8]. The length of the tail rod below the lower connector was recorded serially throughout the follow-up period, beginning from the immediate postoperative X-ray. All of the AVWA values mentioned above were measured by the first author (Y. Z.). A week later, the first author measured the AVWA values picked randomly to evaluate intra-observer reliability. The last author (G.W.) also measured the AVWA values to evaluated inter-observer reliability.

The goal of the study was to identify the amount and rate of change in Cobb angle, AVWA, remaining rod lengths, maximal thoracic kyphosis and total T1-S1 heights after the PRSS surgery during the course of study. The study was approved by the hospital medical ethics committee, and informed consent was obtained for each of the study participants.

### Statistical analysis

Total spine X-rays were performed preoperatively, postoperatively and each follow-up visit for every patient, and measurements described above were recorded and analyzed. The intraclass correlation coefficient (ICC) was used to evaluate inter-observer and intra-observer reliability (ICC ≥ 0.8 was considered to indicate excellent reliability). Differences between two groups were evaluated with unpaired Student’s *t* test. Preoperative and postoperative differences in the same group were evaluated with paired Student’s t test. All statistical analyses were performed by using GraphPad Prism v8.0.2, and *P* < 0.05 was considered statistically significant.

## Results

**Fig. 3:**
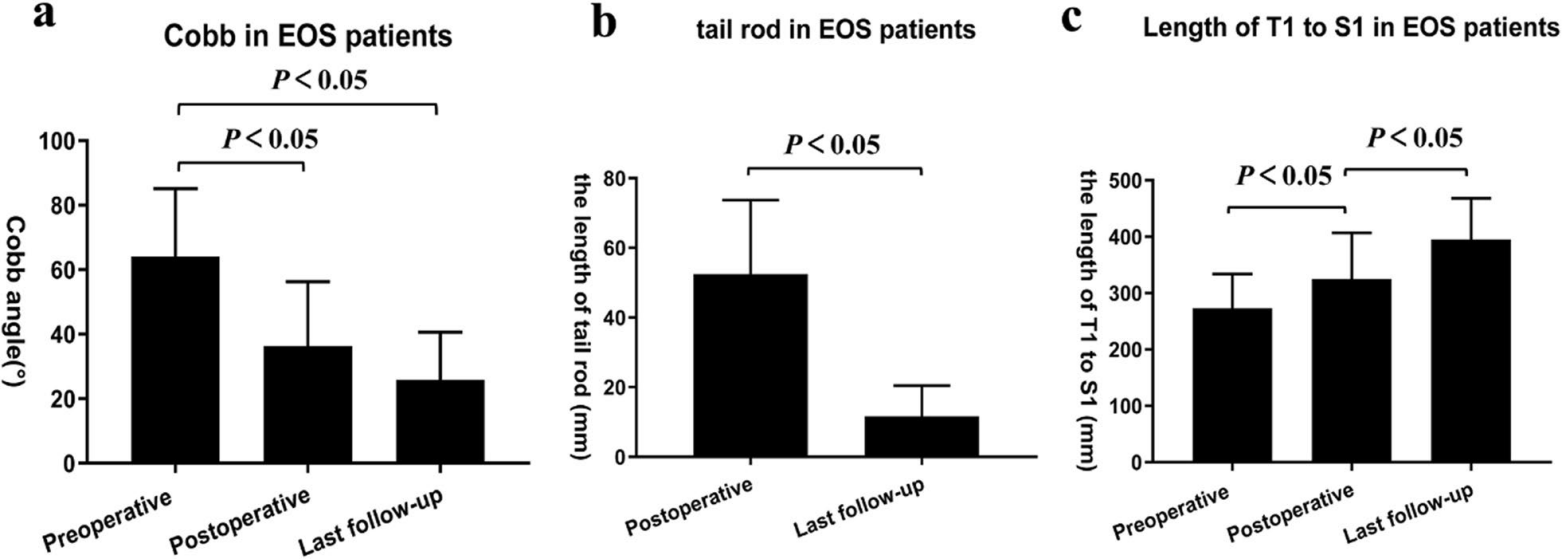
**a** There was significant decrease in the Cobb angle after plate-rod spinal system surgery (preoperative vs postoperative; *P* < 0.05) in children with early onset scoliosis, and significant correction (preoperative vs last follow-up; *P* < 0.05) was maintained during long-term follow-up. (**b**) The length of tail rods were obviously decreased at the last follow-up compared with that of the immediate postoperative (*P* < 0.05). **c** The length of T1 to S1 was significant increase in Cobb angle after plate-rod spinal system surgery (preoperative vs postoperative and postoperative vs last follow-up; *P* < 0.05)

Thirty-one patients, 9 boys and 22 girls, with a mean age of 7 years (range 3–10 years) who had progressive EOS treated by PRSS were completed follow-up. And four patients were lost to follow up. The mean follow-up length was 72 months (range 24–108 months). The mean preoperative Cobb angle was 64° (range, 40°–82°), which was corrected to 36° (range, 10°–41°) immediately after surgery, with an average correction rate of 43.75%. The mean Cobb angle at the last follow-up was 26° (range, 5°–33°) (*P* < 0.05 vs preoperative measurement), with an average correction rate of 59.38% (Table 1 and Fig. 3).

**Table 1:** Summary of radiographical results of all patients at preoperative, postoperative and latest follow-up time points

	Mean preop^a^	Mean postop	Latest follow-up
Cobb angle	64°	36°	26°
AVWA	–	15°	5°
Rod tail reserve length(mm)	–	53	12
Maximal thoracic kyphosis	41°	35°	30°
Total T1-S1 heights (mm)	273	325	395

^a^Preop indicates preoperative; Postop indicates postoperative

The mean tail rod reserve length was 53 mm immediately after surgery and decreased to 12 mm at the last follow-up (*P* < 0.05) (Fig. 3b). The mean preoperative maximum thoracic kyphosis was 41° decreased to 35° and 30° postoperatively and at latest follow-up, respectively (Table 1). The mean preoperative T1–S1 height for all 32 patients was 273 mm and increased to 325 mm (*P* < 0.05) and 395 mm (*P* < 0.05) after initial surgery and latest follow-up, respectively (Fig. 3c).

**Fig. 4:**
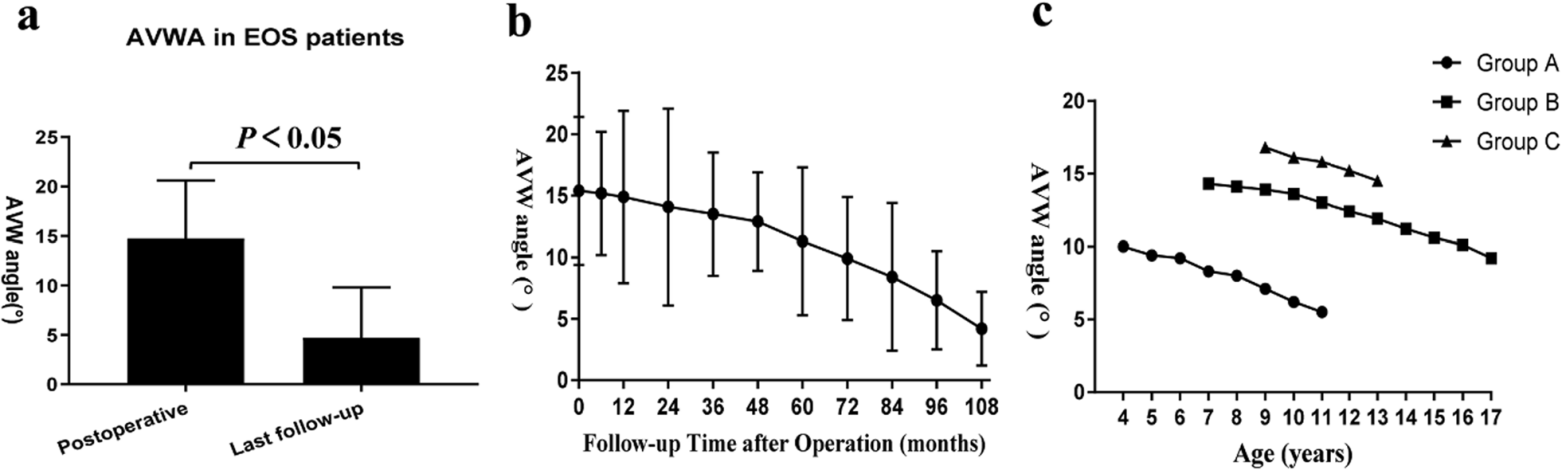
The apical vertebral wedge angle (AVWA) after correction of early onset scoliosis with the plate-rod spinal system. **a** AVWA on the postoperative X-ray compared with AVWA on X-ray at the last follow-up (*P* < 0.05). **b** Etiology of early onset scoliosis and AVWA. In general, the decrease was not obvious during the first two postoperative years, and decreased markedly from years 3–6

**Fig. 5:**
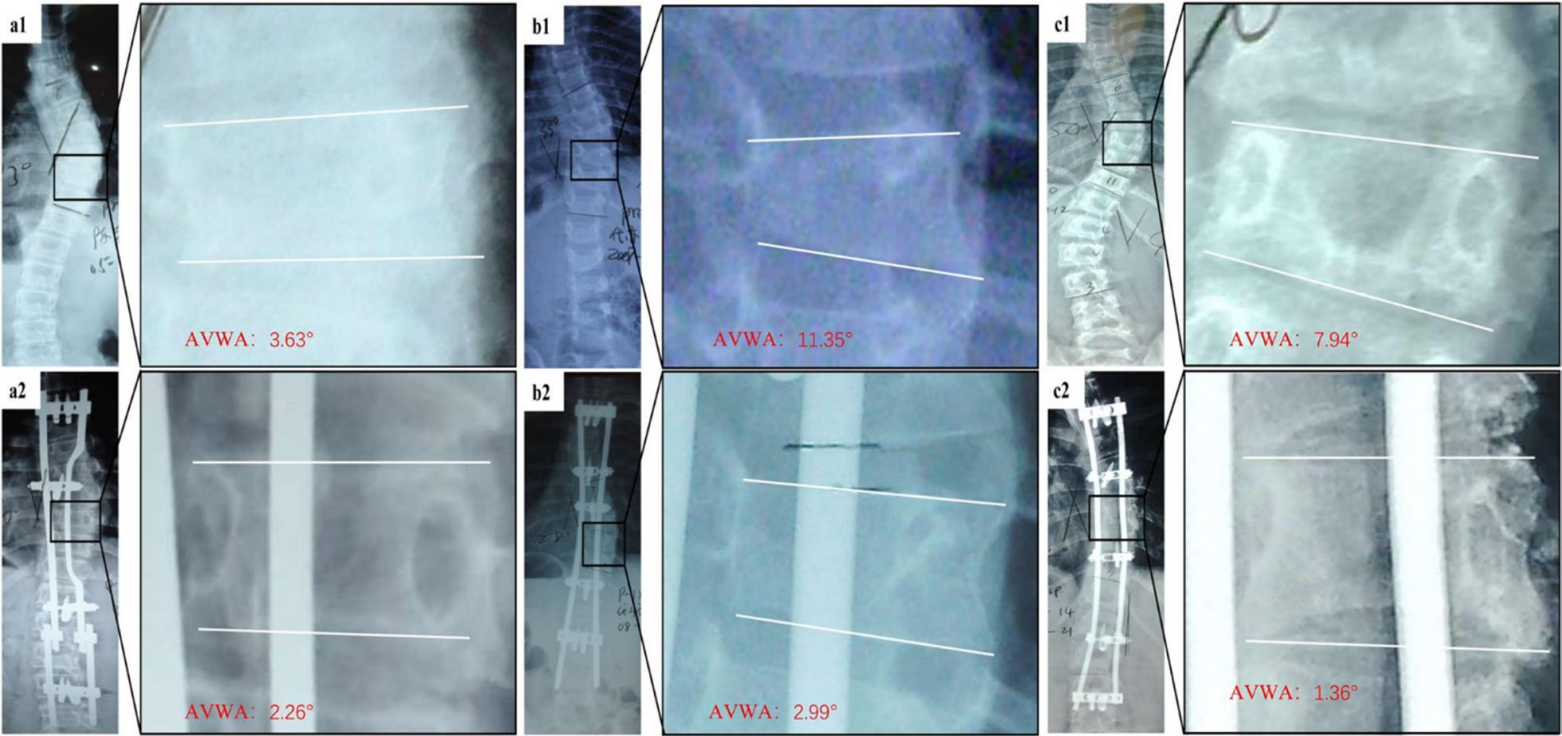
Representative images of long-term changes in the apical vertebral web angle (AVWA) after insertion of plate-rod spinal system in three patients with early onset scoliosis (EOS). **a1**, **a2** An 8-year-old girl with EOS, the postoperative AVWA is 3.63° and the AVWA is 2.26° at postoperative 2 years. **b1**, **b2** A 3-year-old girl with EOS, the postoperative AVWA is 11.35° and the AVWA is 2.99° at postoperative 2 year. **c1**, **c2** A 10-year-old girl with EOS, the postoperative AVWA is 7.94° and the AVWA is 1.36° at postoperative 4 years

Inter-observer reliability in measuring AVWA was excellent with ICCs of 0.976. Intra-observer reliability in measuring AVWA was also excellent with ICCs of 0.984. The average AVWA was 15° immediately after surgery and was decreased to 5° at the last follow-up, and this change was significant (*P* < 0.05) (Table 1). The AVWA showed an overall declining trend during the entire follow-up period. As described in Fig. 4, the decrease was not obvious during the first two postoperative years, and decreased markedly from years 3 to 6 (Fig. 5).

### Complications

There are two complications occurred in two patients through the latest follow-up evaluation. And the two patients had required reoperation. Reasons for reoperation included: rod fracture and rod reached its full lengthening potential. The two patients were treated with replacement of rod. No death, neurological deficit, infection or other complications occurred in other patients.

## Discussion

**Fig. 6:**
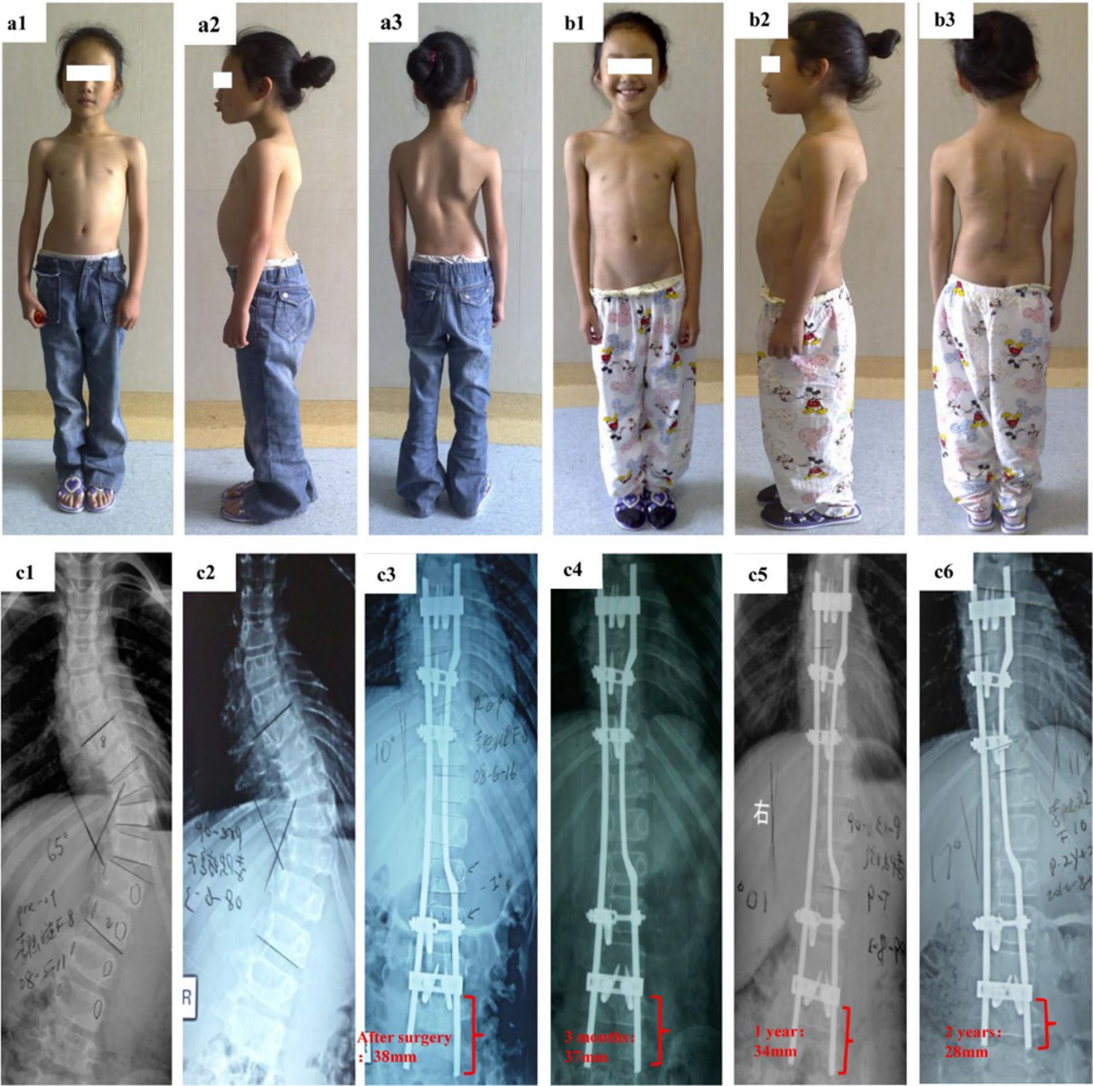
An 8-year-old girl with progressive early onset scoliosis before and after treatment with the plate-rod spinal system. **a1**–**a3** Preoperative photographs. **b1**–**b3** Postoperative photographs. **c1** On plain radiographs, the preoperative Cobb angle is 65° before traction and **c2** 50° after traction. **c3** The postoperative Cobb angle is 10° and the rod tail reserve length is 38 mm. **c4** The rod tail length on plain X-ray at three months after surgery is 37 mm. **c5** and **c6** The rod tail length at one- and two years after surgery is 34 mm and 28 mm

**Fig. 7:**
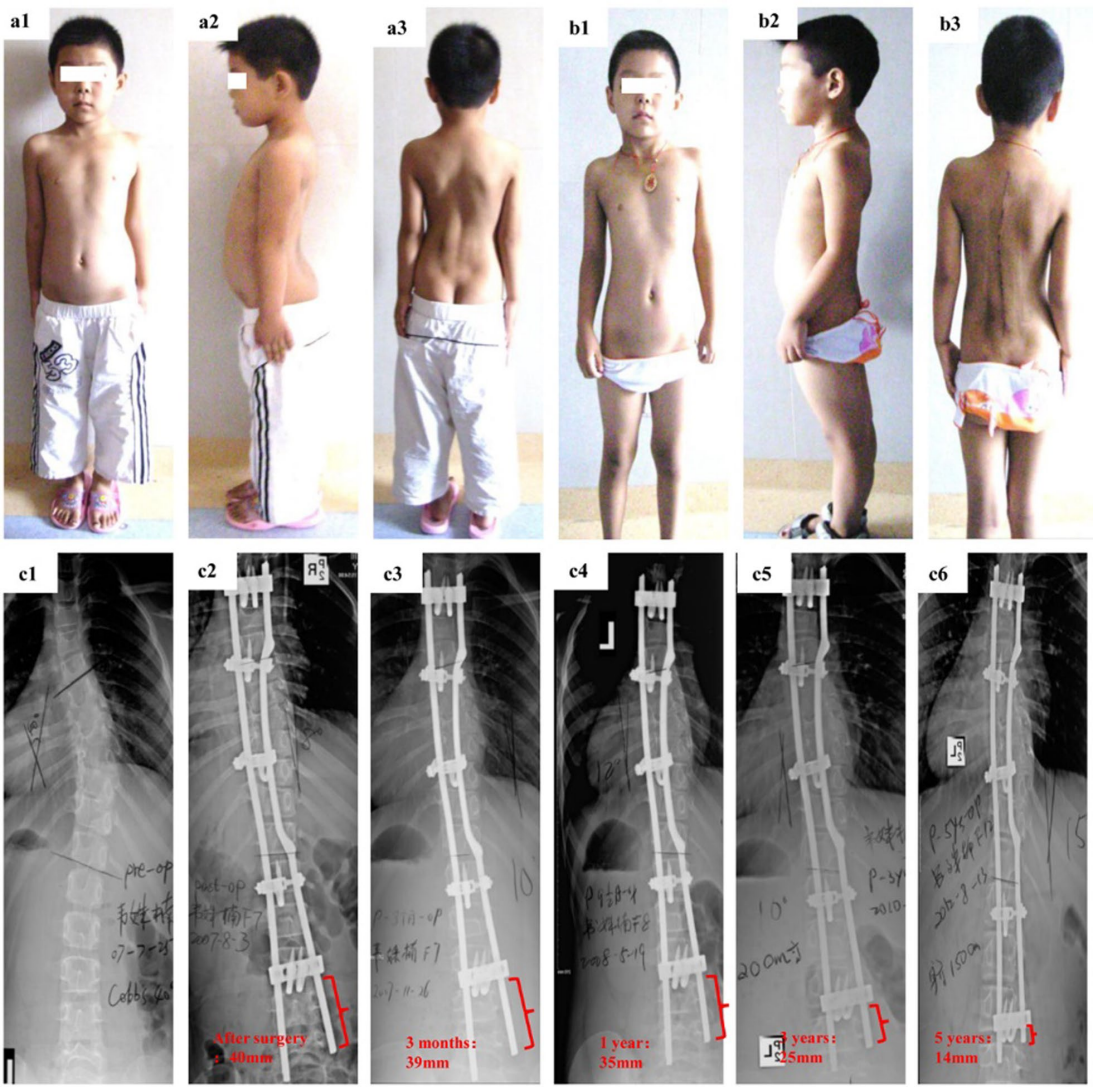
A 5-year-old boy with progressive early onset scoliosis treated by the plate-rod spinal system. **a1**–**a3** Preoperative photographs. **b1**–**b3** Postoperative photographs. **c1**–**c6** On plain radiographs, **c1** the preoperative Cobb angle is 40°. **c2** The postoperative Cobb angle is 8° and the postoperative rod tail reserve length is 40 mm. **c3**–**c6** The rod tail length at 3 months, 1 year, 3 years and 5 years postoperatively is 39 mm, 35 mm, 25 mm and 14 mm

**Fig. 8:**
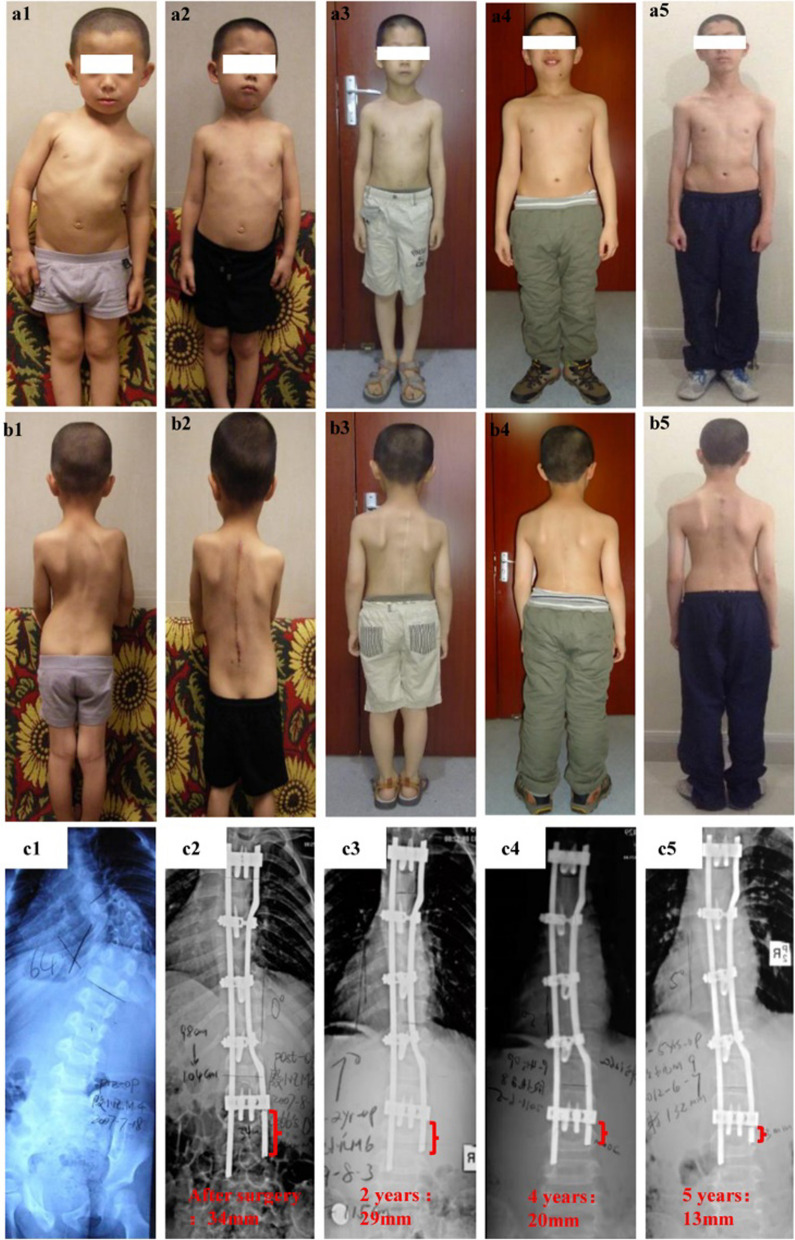
Immediate and ongoing correction of early onset scoliosis (EOS) after treatment with the plate-rod spinal system. **a1**, **b1**, **c1**, **d1** A 4-year-old boy with a preoperative Cobb angle of 64°. **a2**, **b2** Postoperative photographs. The postoperative radiograph **c2**, **d2** shows a Cobb angle of 0° degrees and a rod tail reserve length of 34 mm. **a3**, **b3**, **c3**, **d3** Photographs and radiograph at postoperative 2 years. The rod tail length is 29 mm. **a4**, **b4**, **c4**, **d4** Photographs and radiograph at postoperative year 4. The rod tail length is 20 mm. **a5**, **b5**, **c5**, **d5** Photographs and radiograph at postoperative year 5. The rod tail length is 13 mm

Treatment of EOS with currently available technology is aimed at partially correcting scoliosis and maintaining correction while avoiding fusion and preserving mobility of motion segments. PRSS is a promising technique and early results have shown that it is able to maintain normal growth of the spine and avoid deterioration of the curve. Correction of the spinal deformity occurs at the operative mounting of the PRSS, which creates an asymmetrical stress with the plate-rod pushed in at the convexity (Fig. 1). There was no additional distraction force applied during the operation, which decreased the risk of nerve-stretch injury, and no bony fusion procedures were required. Some surgical methods aimed to correct scoliosis by applying traction on the concave side and pressure on the convex side of the spine. These procedures required fixation at both ends to achieve the desired correction. The PRSS involves applying lateral thrust on the convex side of the spine to correct scoliosis, with the head of the internal fixation system fixed and the tail sliding. Compared to those operative procedures, this surgery applies a smaller stretching force on the concave side of the spine. This is particularly beneficial for patients with a tethered spinal cord, as it reduces the likelihood of experiencing corresponding neurological symptoms. Three examples are given to show that the tail rods migrated upward with the growth of the spinal column (Figs. 6, 7, 8).

The maintenance of almost physiological spinal growth with the use of PRSS is encouraging. The mean T1–S1 height for all 32 patients was 395 mm in the last follow-up with 1 cm/y growth rate. The mean rod tail reserve length decreased by 41 mm at the last follow-up. And a T1-S1 height of 450 mm at skeletal maturity is considered normal [15]. The mean age of the patients was 12 years old in the last follow-up. It is highly likely that the patients T1-S1 heights continue to have increased. Thus, we continue to follow up patients regularly and keep a close watch on T1-S1 heights changes. Physiological growth rates of the immature spine are reported in the literature by Dimeglio as well [16]. The expected spinal growth regarding T1-S1 heights is about 2 cm/y between birth to age 5, 1 cm/y between age 5 to 10, 1.8 cm/y between age 10 to skeletal maturity [16]. Also, Akbarnia [17] reported a T1-S1 heights growth rate of 1.2 cm/y in a series of 23 patients with the use of TGR after an average follow-up of 4.75 years. The spinal growth after implantation of MCGR through follow-up for T1-S1 heights 0.8 cm/y, which is slightly more than growth achieved after TGR [17]. Both the cobb angle and the maximal thoracic kyphosis angle were decreased slowly during follow-up.

Once installed, the PRSS continues to exert a compressive stress on the convex side, thereby exerting a corresponding tensile stress on the concave side. As a result, a period of smooth attenuation of the AVWA was observed in almost all cases, which resulted in the restructuring of the spine curvature during the growing period. Thus, PRSS can continue to rectify the remaining scoliosis deformity during the growth period. The distal portions of the PRSS rods slide freely within their attachments so that repeated instrument-lengthening operations can be avoided during the whole modulating period.

Experiments in animal models have shown that asymmetric loading causes bony asymmetric growth, as described by the Hueter–Volkmann Law. The effects of distraction and compression on the vertebrae have been described by numerous researches among vertebrates including mice, rats, pigs and calves [10–12, 18]. Common to all has been the finding that vertebrae within distraction forces grow faster than those under compression or without distraction. Wedging occurs when the vertebrae are asymmetrically loaded [10]. During growth, both intrinsically and extrinsically generated mechanical stresses act on chondrocytes in the growth plate and the role of mechanical stresses in promoting tissue growth and homeostasis has been strongly demonstrated in the articular cartilages of the major skeletal joints [13].

Vertebral wedging in scoliosis is largely attributed to unsymmetric growth of the endplate. Longitudinal vertebral growth derives from the endochondral ossification of the vertebral growth plate. In scoliosis, normal zoned architectures are observed in the convex side of the growth plate and disorganized architectures in the concave side [19]. The proliferative potential indexes and apoptosis indexes of chondrocytes in the proliferative and hypertrophic zone in the convex side are significantly higher than those in the concave side of the apical vertebral growth plate [20]. After corrective surgery, the wedge deformity of vertebral bodies can show a reshaping potential toward a symmetrical configuration [21]. Reversal of the deformity in EOS after balancing these forces by PRSS is accompanied by an ongoing measurable decrease in the AVWA in serial X-rays (Fig. 8).

We found that the AVWA was significantly reduced at the last follow-up time compared to just postoperative. The AVWA decreased from 15° to 5°, although a change of just 10 degrees for the entire cohort, the clinical significance of this is the vertebral wedging attenuated involved in spinal deformity correction. The mean AVWA decreased smoothly during the first two postoperative years, and decreased markedly from years 3 to 6. Younger EOS patients undergoing PRSS had a smaller AVWA at baseline. Our results suggest a turning point of spine growth and development from rapid to slow in EOS patients at 5 years old, and we had better results with PRSS for EOS in patients who were treated before the age of 5 years.

The results we have observed here are from a single cohort. A multicenter and large sample study will be necessary to evaluate the accuracy of the AVWA measurement method and the long-term efficacy of the PRSS instrumentation.

## Conclusion

In conclusion, our findings highlight the usefulness of single-stage instrumentation with PRSS for immediate and ongoing correction of the deformity in children with EOS during the growth period. Serial measurement of the AVWA serves as a noninvasive method to evaluate the ongoing rectification of the deformity.

## Data Availability

The datasets analyzed during the current study are available from the corresponding author on reasonable request.
